# Retrospective Analysis of Epidemiology of Intracranial and Spinal Tumor in North Western Part of India: 5-Year Observational Study of 1315 Cases

**DOI:** 10.7759/cureus.102353

**Published:** 2026-01-26

**Authors:** Pravin Kumar, Sweta Sinha, Tashbihul Azhar, Ephraim Rebba, Aishwarya Peshattiwar, Bhargav Adari, Gaurav Gautam, Sneha More, Anbarson Sivamoorthy, Jahnaviba Zala

**Affiliations:** 1 Surgical Oncology, All India Institute of Medical Sciences, Patna, Patna, IND; 2 Obstetrics and Gynaecology, Employee State Insurance Company Hospital, Bihta, Patna, IND; 3 Surgery, Osmania Medical College, Hyderabad, IND

**Keywords:** astrocytoma, cns, intracranial tumor, meningioma, spinal tumor

## Abstract

Introduction

Intracranial tumourstumors arise from the brain or its surrounding tissues. Tumors of the central nervous system (CNS) are estimated to be approximately 2% of all new cancers. In India, they constitute about 1.9% of all tumors. Extramedullary lesions, most commonly benign meningioma, account for 70 to 80% of spinal cord tumors. The objective of this study is to characterize histological subtypes, age and gender distribution, and WHO grading of intracranial and spinal tumors in a tertiary care hospital in Jaipur, Rajasthan, India.

Material and method

A retrospective study was conducted at the Department of Surgical Oncology, Sawai Man Singh (SMS) hospital, Jaipur, Rajasthan, a tertiary care hospital, over a period of 5 years. A total of 1315 cases of CNS and spinal neoplasms were diagnosed during this period.

Results

In this study, a total of 1315 cases of CNS and spinal tumors were found. The majority (1199 cases, 91.2%) of the tumors were intracranial, whereas the remaining 116 (8.82%) cases were spinal. Among intracranial tumors, astrocytomas accounted for the largest share in our study, comprising 398 cases (30.27%), mostly seen after the 3rd decade of life, with a mean age of occurrence of 28.44 years. Glioblastoma multiforme forms the largest subtype, constituting 41.96% of all astrocytoma, mostly seen in the 5^th^ or 6^th^ decade of life. Meningioma constitutes the second major group of tumors involving the CNS. Overall, males outnumbered females, the ratio being 1.66:1. However, meningioma is an exception, which was found with higher rates in females (ratio 1:1.37). Among the spinal neoplasms, meningioma was the most common type in our study.

Conclusion

The most common type of intracranial tumor in the present study was astrocytoma, followed by meningioma. The ratio of male: female for CNS tumors was 1.66:1. However, females outnumbered males in the case of meningioma, with a ratio of 1:1.37. Meningioma was the most common type of spinal neoplasm.

## Introduction

Brain and surrounding tissue tumors, known as intracranial neoplasms, form a diverse group within central nervous system (CNS) cancers, encompassing both non-cancerous and cancerous types [1]. These CNS growths make up roughly 2% of newly diagnosed cancers worldwide, a figure consistent across European and non-European groups [2-4]. In India, they account for about 1.9% of all tumors, though rates climb higher in wealthier nations. Brain tumor cases show a bimodal age pattern, peaking in childhood and again between ages 45 and 70 [5-6].

Adult CNS tumors often differ from pediatric ones in location and type. Children’s tumors frequently develop in the posterior fossa, while adults see most in the anterior regions. Only inherited genetic conditions and exposure to ionizing radiation count as confirmed risk factors for primary CNS tumors [4]. Brain metastases are common, too, especially from lung or breast cancers.

Spinal cord tumors, which comprise 2-4% of primary CNS cases, vary widely in cell type and are grouped by position: extradural, intradural-extramedullary, or intradural-intramedullary. Metastases drive most extradural cases. Benign meningiomas dominate extramedullary tumors, representing 70-80% of spinal growths. Among intramedullary types, astrocytomas prevail in kids, while ependymomas are more typical in adults [7].

The objective of this study is to determine the relative frequency of CNS and spinal tumors in the North Western part of India (Jaipur, Rajasthan) according to the WHO classification 2021. This study is totally based on postoperative pathology specimens, and outcomes are not mentioned here.

## Materials and methods

A retrospective study was conducted at the Department of Surgical Oncology, Sawai Man Singh (SMS) hospital, Jaipur, Rajasthan, a tertiary care hospital, over a period of 5 years. Data were collected from pathology department logs of SMS hospital, Santokba Durlabhji Memorial Hospital (SDMH hospital), Bhagwan Mahavir Cancer Hospital (BMCH hospital), and Keshav Joshi (KCJ) diagnostic center, and medical records of all eligible patients over the specified 5-year period were included in this study. A total of 1315 cases of CNS and spinal neoplasms were diagnosed during this period and included in this study.

Inclusion criteria

1) All patients of any age with histopathologically proven intracranial or spinal tumors from the above-mentioned center during the 5-year study period; 2) Both primary and secondary (metastatic) CNS/spinal tumors.

Exclusion criteria

1) Non-neoplastic CNS/spinal lesions such as hematomas, abscesses, granulomas, lesions, congenital/developmental malformations, etc. 2) Cases with inconclusive, inadequate, or missing histopathology.

Each and every patients were explained regarding this study and valid informed consent was taken from each patients and the study was approved by the Institutional Ethics Committee.

Statistical analysis

Data were entered in Microsoft Excel and checked for completeness and consistency before analysis. All statistical analyses were performed using Microsoft Excel (Microsoft Corp.), utilizing built-in functions. Continuous variables (such as age) were summarized as mean and standard deviation. Categorical variables (such as sex, tumor location, and histological type) were presented as frequencies and percentages in tables and charts.

## Results

Out of the 1,315 CNS and spinal tumor cases analyzed, 745 (56.65%) were classified as malignant and 570 (43.35%) as benign (Table 2). CNS tumors accounted for 1,199 cases, while 116 were spinal neoplasms. Patient ages ranged from 1 to 80 years, with a mean age of 27.83 years. The highest occurrence (42%) was in individuals aged between 31 and 60 years. Males were more commonly affected (n = 822; 62.5%) than females (n = 493; 37.5%), resulting in a male-to-female ratio of 1.66:1. However, meningiomas were more frequently diagnosed in females, with a female predominance (1:1.37).

Among intracranial tumors, astrocytomas were the most frequently diagnosed (30.27%, n = 398) (Table 1) and typically appeared after the third decade of life, with an average onset at 28.44 years. Glioblastoma multiforme was the most common astrocytoma subtype (41.96%), mostly affecting individuals in their 50s and 60s. Meningiomas comprised the second-largest group (17.49%, n=230), followed by oligodendrogliomas (6.92%, n=92). Less common tumors, including malignant peripheral nerve sheath tumors (MPNST), neurocytomas, mixed gliomas, hemangioblastomas, and hemangiopericytomas, collectively made up 5.55% (n=73). Brain metastases were detected in 24 cases (1.83%), with adenocarcinoma being the most frequent histological type.

In the spinal group, meningiomas were the most commonly encountered tumor type (1.37%, n=18 of all cases) (Table 1), followed by ependymomas and neurilemmoma, each contributing 1.22% (n=16). Rare tumors, such as neurofibromas, angiomyolipomas, hemangioblastomas, and hemangiopericytoma, constituted 2.89% (n=38) of total cases.

**Table 1:** Gender distribution of different histological types of intracranial and spinal tumors

Histological Type	Male	Female	Total (%)	P-value
Intracranial Tumor				
Astrocytoma	272 (33.17%)	126 (25.45%)	398 (30.27%)	<0.01
Craniopharyngioma	25	14	39 (2.97%)	0.86
Meningioma	99 (12.07%)	131 (26.46%)	230 (17.49%)	<0.001
Ependymoma	19	5	24 (1.83%)	0.09
Oligodendroglioma	63	28	91 (6.92%)	0.17
Metastasis	14	10	24 (1.83%)	0.67
Schwannoma	46	37	83 (6.31%)	0.19
Neurilemmoma	32	31	63 (4.79%)	0.06
Medulloblastoma	50	19	69 (5.25%)	0.09
Non-Hodgkin’s lymphoma	2	5	7 (0.53%)	0.11
Pituitary adenoma	58	29	87 (6.62%)	0.42
Ganglioglioma	4	3	7 (0.53%)	1.0
Ganglioneuroma	2	2	4 (0.30%)	0.63
Miscellaneous	49	24	73 (5.55%)	0.45
Spinal Tumor				
Ependymoma	7	9	16 (1.22%)	0.12
Meningioma	7 (0.85%)	11 (2.22%)	18 (1.37%)	0.04
Astrocytoma	3	0	3 (0.23%)	0.29
Neurilemmoma	16 (1.95%)	0	16 (1.22%)	<0.001
Schwannoma	13 (1.58%)	0	13 (0.99%)	<0.01
Metastasis	10	2	12 (0.91%)	0.22
Miscellaneous	29	9	38 (2.89%)	0.08
Total	820	495	1315 (100%)	

Malignancies were slightly more common than benign lesions, with 745 malignant versus 570 benign tumors (Table 2). Of the malignant cases, 97.26% were primary CNS and spinal tumors, while 2.73% were metastatic. A higher incidence of malignancies was noted in the fourth and fifth decades of life, with a decline in the sixth and seventh decades (Table 2).

**Table 2:** Distribution of patients according to age group

Age Group (years)	Malignant	Benign	Total (%)	p-value
0-10	120	90	210 (15.97%)	0.87
11-20	69 (9.26%)	30 (5.26%)	99 (7.53%)	<0.01
21-30	92	87	179 (13.61%)	0.12
31-40	154	129	283 (21.52%)	0.39
41-50	192	139	331 (25.17%)	0.56
51-60	76	62	138 (10.49%)	0.69
61-70	33	27	60 (4.56%)	0.79
71-80	9	6	15 (1.14%)	1.0
Total	745 (56.65%)	570 (43.35%)	1315 (100%)	

Histologically, Grade I tumors were most prevalent (52.09%, n=685) among all cases (Table 3).

**Table 3:** Frequency of various intracranial and spinal tumors according to WHO grading

WHO Grading	Number of Cases (%)
Grade I	685 (52.09%)
Grade II	116 (8.82%)
Grade III	82 (6.24%)
Grade IV	432 (32.85%)
Total	1315 (100%)

In astrocytomas specifically, Grade IV tumors (glioblastomas) were more common (44.88%) compared to Grade I (17.46%), following the WHO 2021 classification. Overall distribution showed Grade I tumors at 52.09% (n=685), Grade IV at 32.85% (n=432), Grade II at 8.82% (n=116), and Grade III at 6.24% (n=82) (Table 4). Between the age group of 41-60, Grade I cranio-spinal tumors are most common, contributing for 39.88% (n=272).

**Table 4:** The relationship between the distribution of age and the grading of tumor

Age Group (Years)	WHO Grading				
	Grade I	Grade II	Grade III	Grade IV	p-value
0-20	99	20	15	88	0.08
21-40	177	42	26	108	0.06
41-60	272 (39.88%)	34 (29.31%)	24 (29.26%)	135 (31.25%)	<0.01
61-80	137	20	17	101	0.41
Total (1315)	685	116	82	432	

## Discussion

Intracranial tumors are significant neurosurgical conditions due to their potential for high morbidity and mortality. In line with prior studies, our data showed a higher prevalence of brain tumors compared to spinal tumors. In our analysis, we observed a bimodal age distribution with one peak in childhood and another between 31 and 60 years. Out of 1,315 brain and spinal tumor cases, 614 (46.69%) were in the 31-50-year age group, with the highest concentration (331 cases, 25.17%) occurring between ages 41 and 50. These findings are consistent with those reported by Thakur et al. [8]. Male patients were more prevalent overall, with a ratio of 1.66:1, although meningiomas showed a higher occurrence in females, at a ratio of 1:1.37. Similar sex distribution was also noted by Ghanghoria et al. [9-11].

Intracranial tumors represented the vast majority of cases (91.2%), with spinal tumors comprising only 8.82%. Astrocytomas were the predominant tumor type, accounting for 398 cases or 30.27% of all CNS tumors. This aligns with reports from Jalali and Datta, Jibrin et al., Mohammed et al., Le Ruhn et al., who similarly identified astrocytomas as the most common CNS tumor [12-16]. Meningiomas were the second most frequent CNS tumor (230 cases, 17.49%) and also the most common spinal tumor (18 cases, 1.37%), consistent with previous research [17,18]. However, studies by Ghanghoria et al., Das et al., and Lee et al. found meningiomas to be the most common CNS tumors in their cohorts [11,19,20]. Among astrocytic tumors, WHO Grade IV glioblastomas were the major subtype, comprising 41.96% of astrocytomas. Similar trends were observed by Yadav et al., Mondal et al., and Ghanghoria et al. [1,10,11].

Regarding metastatic tumors, adenocarcinoma, papillary carcinoma, and squamous cell carcinoma were the main histologic types spreading to the brain and spinal cord (36 cases), with adenocarcinoma being the most prevalent (21 cases). Colonic adenocarcinoma was the most frequent primary site. Mohammed et al. reported eight metastatic brain tumors, of which 87.5% were adenocarcinomas [17]. We also identified 87 cases (6.62%) of pituitary adenoma, consistent with the findings of Das et al. [19]. Additionally, CNS lymphoma was diagnosed in seven cases (0.53%) and spinal lymphoma in eight cases (0.61%), which aligns with previously reported incidence rates of 0.8% to 1.5% in various studies.

Limitations of our study

As this study was a retrospective study, there was an unavailability of proper history of patients, so we couldn’t draw a conclusion about the etiological association of intracranial and spinal neoplasms. Also, we can’t describe the most common symptoms and the most commonly affected brain site by these tumors. Also, this study lacks postoperative follow-up, so it cannot determine the outcome of these patients.

Our study included only operated cases, excluding patients who underwent upfront radiotherapy. Although Jaipur receives the highest volume of patient load from Rajasthan and nearby states, there are other neuro-oncological centers in the vicinity where patient inflow is much higher. Our study may thus be underestimating the actual incidence of intracranial and spinal tumors in Jaipur, Rajasthan (North Western part of India). Also, this study utilized only histopathologically proven cranio-spinal tumors, so there is a chance of missing some data in this study.

## Conclusions

This retrospective study of 1315 cases intracranial and tumors in the North Western region of India reveals a striking predominance of intracranial neoplasms (91.2%), with astrocytomas emerging as the leading histology (30.27%) and glioblastoma as its aggressive subtype (41.96% of astrocytomas). The bimodal age pattern-peaking in childhood and robustly in the 31-60-year range, especially 41-50 years (25.17% of cases)-mirrors global trends but underscores a heavy burden on India's working-age adults, where males outnumber females (1.66:1 ratio) except for female-skewed meningiomas. Malignant tumors (56.6%) outnumbered benign ones, with WHO grade I lesions most common overall (52.09%) yet grade IV dominating astrocytic cases, highlighting opportunities for early detection and targeted interventions in resource-limited settings to mitigate morbidity in this productive demographic.

Limitations of our study

As this study was retrospective study, there was unavailability of proper history of patients, so we can’t draw conclusion about etiological association of intracranial and spinal neoplasm. Also we can’t describe about the most common symptoms and most common affected brain site by these tumors. Also this study lacks the postoperative follow-up, so can’t determine the outcome of these patients.

Our study included only operated cases excluding patients who underwent upfront radiotherapy.

Although Jaipur receives the highest volume of patients load from Rajasthan and nearby states, still there are other neuro-oncological centre in the vicinity where patient inflow is much more. Our study may thus be underestimating the actual incidence of intracranial and spinal tumor in Jaipur, Rajasthan (North Western part of India). Also this study utilized only histopathologically proven cranio-spinal tumors, so there is chance of missing some data in this study.
